# Self-Management Exercise Regimen Versus Supervised Hand Therapy in Patients With Dorsal Dislocation or Hyperextension Injury With a Volar Plate Avulsion of the Proximal Interphalangeal Joint: The Randomized Controlled PINOT Study

**DOI:** 10.1177/15589447261469595

**Published:** 2026-08-18

**Authors:** Dorien A. Salentijn, Priscilla Jawahier, Marit van Barreveld, Tosca Vrolijk-van Hengel, Niels Schep, Marcel G. W. Dijkgraaf, Mark van Heijl, Ruben N. van Veen

**Affiliations:** 1 Amsterdam UMC, the Netherlands; 2 OLVG, Amsterdam, the Netherlands; 3 Maasstad Ziekenhuis, Rotterdam, the Netherlands; 4 Trialbureau Zorgevaluatie Nederland, Diemen, the Netherlands; 5 Diakonessenhuis, Utrecht, the Netherlands

**Keywords:** volar plate, PIP joint, finger dislocations, MHQ, hand therapy, self-management, randomized controlled trial, health policy, hand

## Abstract

**Background:**

Supervised hand therapy (SHT) with early mobilization is recommended for patients with a stable, congruent proximal interphalangeal (PIP) joint after dorsal dislocation or hyperextension injury with a volar plate avulsion. Early mobilization uses a dorsal block splint. A self-management exercise regimen (SMER) may offer a valuable alternative that reduces demand on therapists. We hypothesized that SMER is noninferior to SHT.

**Methods:**

Noninferiority was assessed in adult patients in a multicenter, open randomized trial. Noninferiority to SHT had to be shown in both the modified intention-to-treat (mITT) and per-protocol (PP) analysis sets. The predefined noninferiority margin was −8 points, about half the minimal clinically important difference, with a 1-sided critical *P* value of .025.

**Results:**

The mITT analysis set contained 100 patients in each treatment group. The mean difference at 3 months in Michigan Hand Outcomes Questionnaire (MHQ) change score from baseline was −0.6 (95% CI, −4.4 to 3.2; *P*_noninferiority_ = .0001) against SMER. The PP analysis set consisted of 85 patients who followed SMER and 79 patients who received SHT. The mean difference at 3 months in MHQ change score from baseline was 0.4 (95% CI, −3.5 to 4.4; *P*_noninferiority_ = .000026) in favor of SMER. No safety issues following SMER were observed.

**Conclusions:**

The randomized controlled trial showed that SMER for patients with a stable and congruent joint alignment after dorsal dislocation or hyperextension injury with a volar plate avulsion fracture of the PIP joint is clinically noninferior regarding hand function compared with SHT.

**Level of evidence:**

I

## Introduction

An evidence-based treatment strategy is lacking for patients with dorsal dislocations or hyperextension injuries of the proximal interphalangeal (PIP) joint.^1^ The incidence of these finger injuries is approximately 10 000 a year in the Netherlands. They frequently occur in the context of ball sports and are typically attributed to hyperextension or axial loading mechanisms.^2,3^ Most patients preserve a stable and congruent joint alignment after the trauma. A stable joint permits early mobilization using a dorsal block splint.^4^

Previous research has shown that early active motion within a dorsal block splint allowing full extension results in better range of motion than static splinting.^5^ The Dutch guideline recommends that all patients with these injuries seek referral to a hand therapist within 1 week.^1^ Substantial clinical evidence in support of superiority of such supervised hand therapy (SHT) over a self-management exercise regimen (SMER) is unavailable. Self-management exercise regimen is expected to be less costly than SHT and may be a valuable alternative. The aim of the PINOT study is to determine whether an SMER is at least as effective as SHT concerning outcome in hand function for adult patients with a dislocation or hyperextension injury of the PIP joint. Our hypothesis was that hand function after SMER is as good as hand function after SHT at 3 months post-trauma.

## Materials and Methods

### Design and Setting

The PINOT study was a prospective, multicenter, open randomized clinical noninferiority trial comparing SMER (intervention group) with SHT (control group) regarding hand function in adult patients with volar plate injuries who presented at the emergency department (ED) in 4 Dutch peripheral treatment centers. The choice of hospitals was made intentionally, as volar plate injuries are most commonly treated in peripheral hospitals rather than in academic centers.

Trial progress was assessed by independent monitors of Trialbureau Health Care Evaluation Netherlands and TAPAS Group B.V. Netherlands.

The study was conducted in accordance with the CONSORT (Consolidated Standards of Reporting Trials) guidelines for randomized controlled trials.^6^ Written informed consent was obtained from all participants prior to enrollment in the study.

### Participants

Consecutive patients presenting at the ED with a dorsal PIP joint dislocation or hyperextension injury were screened for eligibility (Table 1). Eligible patients were approached by a physician for the informed consent procedure.

**Table 1.** Inclusion/Exclusion Criteria.

Inclusion criteria
• Adult (aged ≥18 years) • Presenting at the ED with ○ Complete dorsal dislocation, confirmed by a medical professional/radiographic imaging at the ED or ○ Volar plate rupture type I, II, IIIa by the standardized Eaton classification of the second to fifth ray, confirmed by an avulsion fracture involving <40% of the proximal volar part of the middle phalanx on a lateral radiograph at the ED • Initial treatment at or within 48 hours of the ED visit with a dorsal block splint in 0° of extension for a duration of 4 weeks conforming the recommendation following the Dutch guideline^1^
Exclusion criteria
• Surgical treatment • Irreducible dislocations • Re-dislocation during digit motion • Unstable joint following reduction as assessed by dorsal, volar, and lateral stress on the PIP joint in extension and 30° of PIP joint flexion • Suspicion for interposition of the volar plate • Multiple injuries of the affected and contralateral hand or extremity • Multi trauma patients (injury severity score ≥16) • Impaired hand function prior to injury disorders of bone metabolism other than osteoporosis or connective tissue diseases • Insufficient comprehension of the Dutch language

Abbreviations: ED, emergency department; PIP, proximal interphalangeal.

### Randomization

Within 1 week following the date of trauma, revisiting eligible patients who gave informed consent were randomized in a 1:1 ratio to SMER (intervention) or SHT (control) in random blocks of different sizes and stratified by type of injury (dorsal dislocation or hyperextension) and age (18-34, 35-64, ≥65) to avoid imbalances between treatment groups. Given the nature of the interventions, blinding of patients was unfeasible. Randomization was performed by the treating physician using Castor EDC.

### Interventions

The intervention group received well-defined SMER instructions to allow starting exercises within 1 week following injury (Supplemental Figure S1). Supervised hand therapy as the control treatment also started within 1 week after injury. Supervising hand therapists were briefed in advance and followed a standard protocol with the frequency of therapies offered to a patient left at their discretion. Both groups were free to perform exercises at home with the device.

### Outcomes and Data Collection

The primary outcome was defined as change in hand function between baseline and 3 months after trauma. Hand function was measured at baseline, 6 weeks, and 3 months using the validated Michigan Hand Outcomes Questionnaire (MHQ).^7,8^ The MHQ is a questionnaire divided into 6 subscales: overall hand function, activities of daily living, work performance, pain, aesthetics, and satisfaction with hand function.^9^ For the pain subscale, higher scores indicate more pain; in the other 5 subscales, higher scores denote better hand performance. The normalized final MHQ score is a summation of the subscale scores (while converting the pain subscale score) divided by 6 and ranges from 0 (severe disability) to 100 (no disability).^9^

Secondary outcomes included active range of motion, pain, and patient expectation for level of recovery.

The total active range of motion (TAM) was determined at baseline, 6 weeks, and 3 months following assessment with a goniometer of the active range of motion of the metacarpophalangeal joint, the PIP joint, and the distal interphalangeal (DIP) joint minus any extension deficits.^10,11^ Both the injured finger and the contralateral finger were measured, and the range of motion was presented as percentage of the injured finger compared with the uninjured one. Pain was assessed at baseline, 6 weeks, and 3 months with a visual analogue scale (VAS) with 0 signifying no pain and 100 representing most severe pain. Patients’ expectations for level of recovery were assessed at baseline using a 5-point Likert scale: 1, no improvement, full restriction; 2, slight improvement, serious restriction; 3, moderate improvement, moderate restriction; 4, substantial improvement, slight restriction; 5, complete improvement, no restriction. Achievement of expectation was assessed at 3 months with the same Likert scale.

### Sample Size and Noninferiority Margin

We hypothesized an at least equal change in MHQ for SMER compared with SHT following the noninferiority study design. We aimed to randomize 222 patients (111 per treatment group, accounting for 5%-10% dropout) to achieve 80% power at 1-sided significance level of .025 with a standard deviation of 20 and setting our noninferiority margin at −8. The noninferiority margin of −8 was based on an estimated minimal clinically important difference (MCID) of 15 points,^12,13^ divided by 2 and rounded up.^14^ The MCID of 15 is based on the average of the MCIDs of 23, 13, and 8 for the pain, function, and work domains, respectively, in patients with carpal tunnel syndrome (CTS).^13^ The absence of an MCID for patients with finger complaints forced us to use the MCID of patients with CTS.

### Statistical Analysis

#### Analysis Sets

Three analysis sets were defined. All randomized patients, following informed consent, constituted the *intention-to-treat* analysis set. Patients who withdrew informed consent and who dropped out early without any clinical follow-up data were excluded.^15^ Another reason for exclusion was treatment by a nonparticipating hand therapist. The remaining patients constituted the *modified intention-to-treat* (mITT) analysis set. Patients in the mITT analysis set were included in the *per-protocol* (PP) analysis set if the treating physician initiated the randomized treatment at baseline and if neither the patient nor the treating physician deviated from the treatment protocol. For patients randomized to SMER, the treating physician determined the end-of-treatment moment or adherence to treatment protocol during follow-up. Any protocol deviation, initiated either by the patient or by the treating physician, such as an outside referral to a hand therapist, resulted in noncompletion of the protocol, with the patient remaining in the mITT analysis set. For patients assigned to SHT, the decision on treatment completion was made by the hand therapist. Patients who dropped out against the advice of the hand therapist were considered not to have completed the protocol and also remained in the mITT analysis set.

#### Data Analysis and Handling of Missing Scores

General descriptive statistics of patient characteristics at baseline were reported for the mITT analysis set as counts and percentages for categorical variables and as means with standard deviation (SD) or medians with interquartile ranges or modes for continuous/discrete variables, depending on data distributions. The baseline characteristics of the ITT and PP analysis sets are reported in Supplemental Tables S1 and S2.

Noninferiority concerning the mean change in MHQ score after 3 months from baseline in the SMER group compared with the mean change in MHQ in the SHT group was analyzed by assessing whether or not the lower limit of the 2-sided 95% confidence interval (CI) of the difference in change scores between SMER and SHT remained within the predetermined noninferiority margin of −8 points, taking SHT as the reference treatment. The corresponding *P* value for noninferiority (*P*_noninferiority_) was derived following independent samples *t* tests, after adding the absolute width of the noninferiority margin (|−8|) to the scores of patients in the SMER group. Given the noninferiority design, primary outcome data were tested using both the mITT and the PP analysis sets.^16^ Noninferiority should be demonstrated in both the analysis sets. The possibility of informative censoring in the PP analysis set was examined in case of discrepant results.^17^ Missing MHQ scores were multiply imputed (n = 10) following predictive mean matching, using baseline characteristics and available MHQ scores over time. Means, mean change scores, and mean differences in changes scores, along with their 95% Cis, were pooled over imputation sets and accounted for variation between the multiple imputations.

Secondary outcomes such as TAM, pain VAS, and the level of fulfillment of patients’ expectations were assessed in the mITT analysis set only and descriptively reported for the SMER and SHT groups based on available data. Data are available from the corresponding author upon reasonable request.

## Results

### Participants

**Figure 1.**
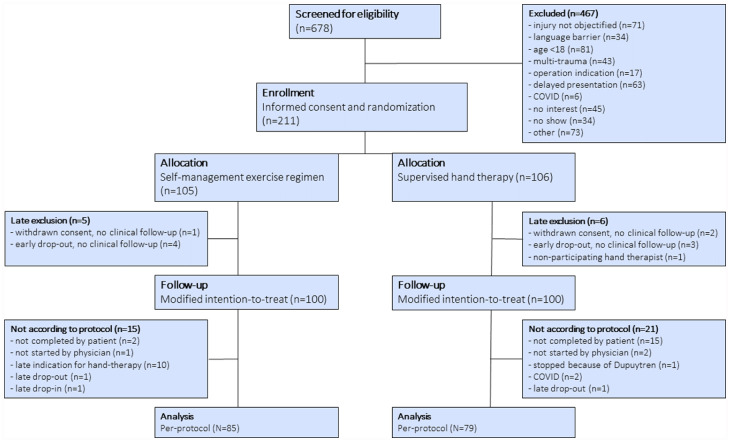
CONSORT (Consolidated Standards of Reporting Trials) flowchart PINOT study.

Between December 1, 2019 and June 1, 2022, at least 678 patients were screened, and, in total, 211 patients were randomized (105 to SMER and 106 to SHT). Eleven patients were excluded after randomization because of withdrawal of consent or lack of follow-up data, resulting in 100 patients per treatment group in the mITT analysis set. Protocol deviations occurred in 15 SMER and 21 SHT patients, leaving 85 and 79 patients, respectively, in the PP analysis set. The study flowchart is presented in Figure 1. The ‘late drop-in’ patient in the SMER group temporarily dropped out, but returned.

Baseline characteristics of the 200 patients in the mITT analysis set are reported in Table 2. Some underrepresentation of female gender and overrepresentation of alcohol use in the SMER group fell short of significance; no major differences were noted. Because of logistics, inclusion at site IV was prematurely closed. Comorbid conditions were infrequent in both groups. A few patients reported prior operations involving either the affected finger (n = 1) or the contralateral finger (n = 5). Inadequate individual ability to access, understand, and use health-related information and services to make appropriate health decisions was limited and comparable for both groups.

**Table 2.** Baseline Characteristics in the mITT Analysis Set per Treatment Group.

Characteristics	Self-management exercise regimen (n = 100)	Supervised hand therapy (n = 100)
Age, y		
18-34	52 (52)	52 (52)
35-64	41 (41)	41 (41)
≥65	7 (7)	7 (7)
Gender (female)	35 (35)	44 (44)
Type of injury (complete dorsal dislocation)	41 (41)	40 (40)
Injured hand (right)	48 (48)	52 (52)
Dominant hand (right)	85 (85)	90 (90)
Dominant hand injured	47 (47)	48 (48)
Cause of injury		
Sports	50 (50)	48 (48)
Work	5 (5)	4 (4)
Other	45 (45)	48 (48)
Study site		
I	59 (59)	62 (62)
II	28 (28)	23 (23)
III	13 (13)	14 (15)
IV	0 (0)	1 (1)
Parkinson	0 (0)	0 (0)
Osteoporosis	4 (4)	1 (1)
Diabetes	3 (3)	2 (2)
Carpal tunnel syndrome (untreated)	0 (0)	2 (2)
History of operations of the affected finger	0 (0)	1 (1)
History of operations of the contralateral finger	2 (2)	3 (3)
Smoker	18 (18)	21 (21)
Alcoholism	32 (32)	20 (20)
Chronic use of corticosteroids	0 (0)	2 (2)
Chronic use of vitamin D	21 (21)	19 (19)
Chronic use of calcium supplements	4 (4)	4 (4)
Chronic use of bisphosphonates	0 (0)	1 (1)
Hormone supplement use	5 (5)	3 (3)
Health literacy (inadequate)^a^	7 (7)	10 (11)
MHQ score, pooled mean (SD)^b^	68.0 (14.9)	67.6 (14.1)

Abbreviations: mITT, modified intention-to-treat; MHQ, Michigan Hand Outcomes Questionnaire.

Count (%), unless otherwise stated.

a N_SMER_N_SHT_ 95.95.

b Originally available: N_SMER_N_SHT_100.99.

The mITT analysis set resembled the ITT analysis set (Supplemental Table S1) regarding distribution of baseline characteristics. The same holds for the mITT and PP analysis sets, with no substantial shifts in site representation (*P* = .68).

Baseline mean MHQ score was 68.0 (pooled 95% CI, 65.0-70.9) in the SMER group and 67.6 (pooled 95% CI, 64.8-70.4) in the SHT group of the mITT analysis set. In the PP analysis set (Supplemental Table S2), baseline mean MHQ score was 68.5 (95% CI, 65.4-71.7) in the SMER group and 67.9 (95% CI, 65.0-70.8) in the SHT group.

### Interventions

The instruction form was given to all patients in the SMER group and all but 1 patient in the SHT group.

In the SMER group, treatment was not initiated by the physician in 1 patient. Treatment according to protocol was completed by 85 patients (85%), of whom 62% (53/85) within 6 weeks and 93% (79/85) within 3 months. During the first 6 weeks, the patients in the SMER group reported practicing, on average, nearly 7 times a day. Five patients (5%) did not practice at all and 46 (46%) practiced at least 8 times a day. During the remainder of the first 3 months, 62.4% of patients reported continuing practicing at a mode (N = 14) of 3 times a day.

In the SHT group, treatment was not initiated by the physician in 2 patients; 79 patients (79%) completed the protocol, of whom 56% (44/79) within 6 weeks and 84% (66/79) within 3 months. During the first 6 weeks, the patients in the SHT group practiced, on average, 6.0 times a day. Ten patients (10%) did not practice at all and 33 (33%) practiced at least 8 times a day. During the remainder of first 3 months, 62.9% of patients reported continuing practicing, also at a mode (N = 20) of 3 times a day.

### Registered Complications

At 6 weeks after trauma, 8 of 99 patients (8%) in the SMER group experienced stiffness, 1 patient (1%) experienced (9°) extension restriction, and 1 patient (1%) had a superficial wound infection and was intravenously treated with antibiotics. At 3 months, 9 of 96 (9%) experience stiffness, 1 patient had a deformity of the position of the finger, and 1 patient’s PIP joint had swollen. No neurovascular or tendon damage was observed in the SMER group.

At 6 weeks after trauma, 8 of 99 patients (8%) in the SHT group experienced stiffness. Two patients (2%) experienced minimal (2°) and mild (6°) extension restriction, whereas 1 patient (1%) had a flexion position of 15°; all 3 were treated with a night splint. At 3 months, 7 of 99 patients (7%) experienced stiffness, and 1 patient (1%) experienced flexion restrictions of the DIP joint (20°) and PIP joint (10°). Two patients had a mild (4°-5°) extension restriction, one of whom received a night splint. No neurovascular or tendon damage was observed in the SHT group.

### Functional Outcome

**Figure 2.**
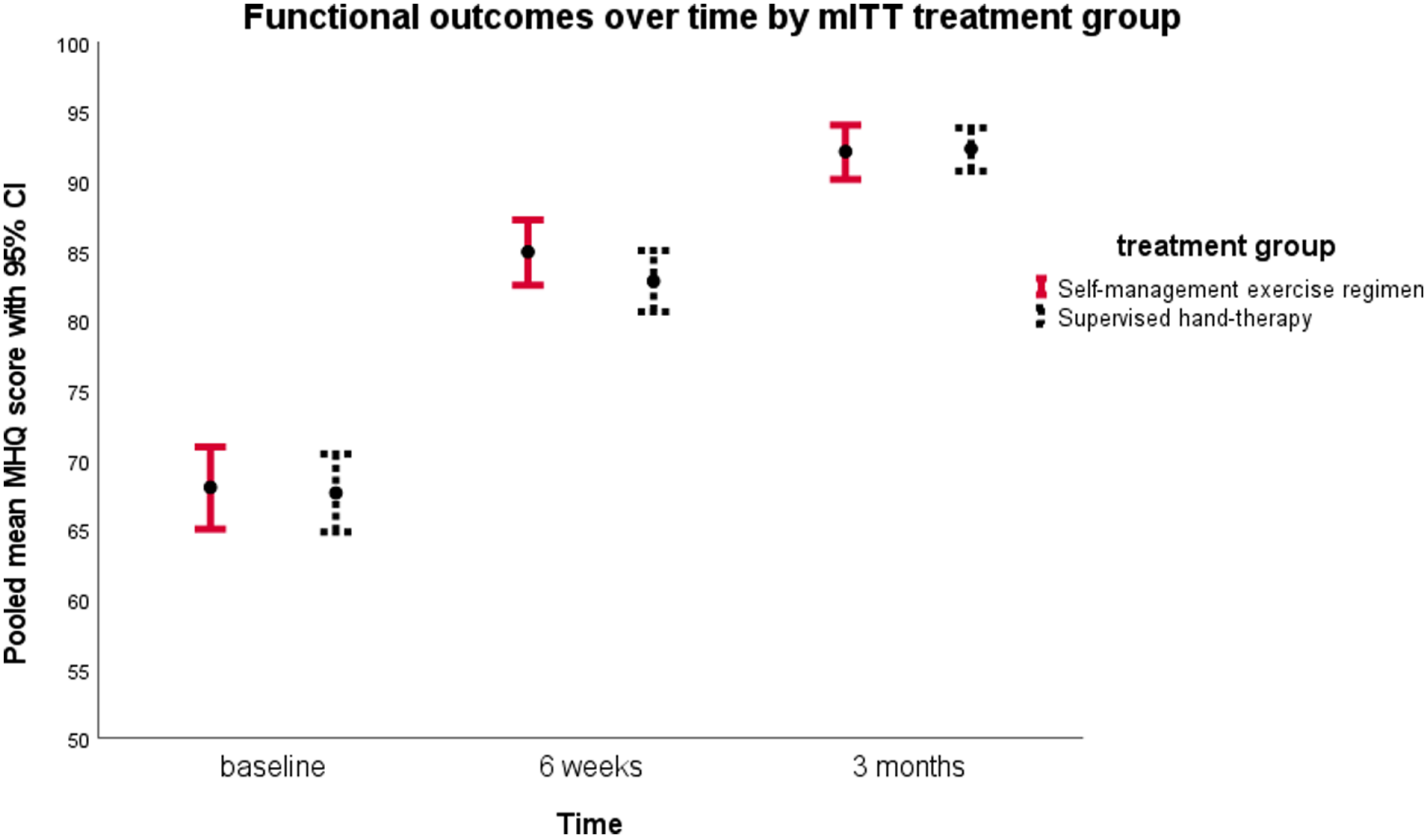
Mean MHQ scores at baseline, 6 weeks, and 3 months by mITT treatment group. mITT indicates modified intention-to-treat; MHQ, Michigan Hand Outcomes Questionnaire.

Mean overall hand function scores based on the MHQ by mITT treatment group are shown in Figure 2. The mean MHQ score increased in the SMER group (N = 100) from 68.0 at baseline to 84.9 (pooled 95% CI, 82.5-87.2) at 6 weeks to 92.1 (pooled 95% CI, 90.1-94.0) at 3 months. The mean change in MHQ score at 3 months compared with baseline was 24.1 (pooled 95% CI, 21.4-26.8). The mean MHQ score increased in the SHT group (N = 100) from 67.6 at baseline via 82.8 (pooled 95% CI, 80.6-85.0) at 6 weeks to 92.3 (pooled 95% CI, 90.7-93.8) at 3 months; the mean change at 3 months compared with baseline was 24.7 (pooled 95% CI, 22.0-27.3). The mean difference in MHQ change score at 3 months between SMER and SHT was −0.6 (pooled 95% CI, −4.4 to 3.2) against SMER. The lower limit of the CI falls within the noninferiority margin of −8 (*P*_noninferiority_ = .0001).

The mean overall hand function scores over time by PP treatment group are shown in Supplemental Figure S2. The mean MHQ score increased in the SMER group (N = 85) from 68.5 at baseline via 86.1 (pooled 95% CI, 83.6-88.5) at 6 weeks to 93.5 (pooled 95% CI, 91.7-95.3) at 3 months. The mean change in MHQ score at 3 months compared with baseline was 25.0 (pooled 95% CI, 22.2-27.7). The mean MHQ score increased in the SHT group (N = 79) from 67.9 at baseline via 83.3 (pooled 95% CI, 80.8-85.9) at 6 weeks to 92.4 (pooled 95% CI, 90.6-94.2) at 3 months; the mean change at 3 months compared with baseline was 24.5 (pooled 95% CI, 21.7-27.3). The mean difference in MHQ change score at 3 months between SMER and SHT was 0.4 (pooled 95% CI, −3.5 to 4.4) in favor of SMER. The lower limit of the CI falls within the noninferiority margin of −8 (*P*_noninferiority_ = .000026).

### Total Active Range of Motion

Based on available data in the mITT analysis set, the mean TAM of the injured finger in the SMER group was 167 (95% CI, 158-177) at baseline (N = 99), 240 (95% CI, 233-247) at 6 weeks (N = 96), and 254 (95% CI, 247-261) at 3 months (N = 87). The TAM as percentage of the contralateral uninjured finger was 64% (95% CI, 60%-67%), 91% (95% CI, 89%-93%), and 95% (95% CI, 93%-96%).

The mean TAM of the injured finger in the SHT group was 162 (95% CI, 152-172) at baseline (N = 100), 240 (95% CI, 233-247) at 6 weeks (N = 94), and 255 (95% CI, 250-260) at 3 months (N-82). The range of motion as percentage of the contralateral uninjured finger was 62% (95% CI, 58%-66%), 90% (95% CI, 88%-93%), and 95% (95% CI, 94%-97%).

### Pain

The mean level of pain at baseline, 6 weeks, and 3 months was 72 (95% CI, 67-77; N = 98), 17 (95% CI, 14-21; N = 94), and 12 (95% CI, 8-15; N = 90), respectively, in the SMER group, and 73 (95% CI, 68-78; N = 97), 18 (95% CI, 14-22; N = 94), and 16 (95% CI, 11-21; N = 88), respectively, in the SHT group.

### Level of Recovery

Patients in the SMER group (N = 89) reported the level of recovery at 3 months. More than half of them recovered as expected (52.8%) or more (10.1%). Substantial improvement with slight restrictions was experienced by 37.1% of patients, whereas 44.9% experienced complete improvement and no restrictions. In the SHT group (N = 88), 48.9% recovered as expected, and 12.4% even more. Substantial improvement with slight restrictions was experienced by 37.5% of patients, whereas 45.5% experienced complete improvement and no restrictions.

## Discussion

This randomized clinical trial aims to show that the SMER for patients with a stable and congruent joint alignment after dislocation or hyperextension injury with a volar plate avulsion fracture of the PIP joint and using a dorsal block splint is clinically noninferior regarding hand function compared with SHT.

Patient-reported outcome scores (measured by the MHQ) at 3 months postinjury were above 90 points in both groups. A recently published Dutch multicenter cohort study, including patients with hand fractures and dislocation, showed that patients receiving SHT had a lower average MHQ score at 3 months compared with patients who did not receive SHT (74 vs 82, respectively).^18^ These findings may support the suggestion that patient responsibility in performing exercises independently can lead to comparable or even better outcomes. This assumption is further supported by a systematic review concerning surgically treated hand fractures which concluded that a well-developed and properly instructed home exercise program is as effective as, or even more effective than, SHT.^19^

Concerning secondary outcomes such as TAM, pain, and level of recovery, we also observed congruent results between treatment groups. For both treatment groups, most patients indicated that their affected hand already had (substantially) recovered within the 3 months after injury. A randomized controlled trial evaluating a home exercise program versus traditional supervised therapy in patients with metacarpal fractures found that, at 3 months postoperatively, the TAM was significantly greater in the home exercise group (256°) compared with the SHT group (245°).^20^ Another randomized controlled trial evaluating patients after surgical treatment of proximal phalangeal fractures found that the supervised rehabilitation program did not lead to better outcomes than without it, in terms of TAM and pain at 6 weeks and 3 months postoperatively.^21^

Clinically, SMER and SHT seem largely interchangeable ‘twin’ treatments, except for patients who initially present with a medical indication requiring professional supervision. This leaves room for further research into patient subgroups that are more or less capable of benefiting from SMER. This issue is addressed in the companion paper on the cost-effectiveness of SMER compared with SHT.

Concerning the type of (im)mobilization material, the dorsal block splint was standardly applied in both groups in addition to the SMER or SHT treatment. However, other types of (im)mobilization materials are available. A recently published randomized controlled trial compared a dorsal block splint (starting at 30° of flexion and gradually increasing to 0° over 4 weeks) with a figure-of-8 orthosis (maintained at 15°-20° for 4 weeks).^22^ The 2 cohorts showed similar outcomes regarding DIP flexion, PIP flexion, pain levels, and overall function. Patients treated with a figure-of-8 orthosis required 4 SHT sessions, compared with 6 sessions for those using a dorsal block splint. These findings suggest that the choice of immobilization material is secondary to the selection of appropriate aftercare. With a properly implemented SMER protocol, the number of required sessions, currently 4 or even 6, could potentially be reduced to 0.

Regarding possible differences in the degree of positioning of a dorsal block splint, the PINOT study used a splint positioned directly at 0°, as supported by the literature.^23^ This approach eliminates the need, in the vast majority of cases, for follow-up visits to the hospital or therapist for splint adjustments. As a result, patients are spared unnecessary travel time, while this practice also helps reduce the burden on an increasingly strained health care system and supports efforts to address the ongoing climate crisis.^24-26^

Reassuringly, the SMER did not appear to increase patient vulnerability or lead to unsafe circumstances. Registered complications were mild and consisted of commonly observed adverse events associated with the trauma in question. This study shows that if patients are able to perform exercises at home following SMER, if their immobilization material is sufficient for the 4-week treatment period, and if their MHQ scores completed from home at 6 weeks and 3 months demonstrate the recovery course as expected, one could even question whether patients with this type of injury still need to return to the hospital for follow-up visits, unless they experience specific complaints. These treatment adaptations toward self-care management have already proven effective in other types of injuries.^27-29^

### Limitations

The study period coincided with the COVID-19 pandemic lockdown.^30^ During this time, patient presentations in trauma care decreased substantially overall.^31^ Regarding solitary hand injuries, finger dislocations accounted for 16% of all cases during the lockdown, compared with 10% in the same period the year before. In absolute numbers, the incidence was more than halved.^32^ It remains unclear whether fewer injuries actually occurred, or whether patients considered the risk of a hospital visit greater than the inconvenience of a finger dislocation. As a result, the inclusion of consecutive patients may have been disrupted, with possibly only more severe cases or younger patients presenting to the ED. However, even if this occurred, the influence on the study results is not expected to be significant. This is because stratification was applied between more complex complete dislocations and minor hyperextension injuries with volar plate rupture, as well as across age groups, ensuring equal representation per treatment arm.

The results of the mITT (N = 200) and PP (N = 164) analysis sets were concordant and robust. This is important because several patients in the SMER group were ultimately advised by their treating physicians to contact a hand therapist for further or faster recovery. This may well be justifiable from a medical point of view, but facilitates demonstrating noninferiority of SMER and is a methodological weakness of the mITT analysis set. However, having demonstrated noninferiority of SMER in the PP analysis set as well underscores that the SMER can be a treatment of first choice for many patients.

## Supplemental Material

sj-docx-1-han-10.1177_15589447261469595 – Supplemental material for Self-Management Exercise Regimen Versus Supervised Hand Therapy in Patients With Dorsal Dislocation or Hyperextension Injury With a Volar Plate Avulsion of the Proximal Interphalangeal Joint: The Randomized Controlled PINOT Study Supplemental material, sj-docx-1-han-10.1177_15589447261469595 for Self-Management Exercise Regimen Versus Supervised Hand Therapy in Patients With Dorsal Dislocation or Hyperextension Injury With a Volar Plate Avulsion of the Proximal Interphalangeal Joint: The Randomized Controlled PINOT Study by Dorien A. Salentijn, Priscilla Jawahier, Marit van Barreveld, Tosca Vrolijk-van Hengel, Niels Schep, Marcel G. W. Dijkgraaf, Mark van Heijl and Ruben N. van Veen in HAND

sj-docx-2-han-10.1177_15589447261469595 – Supplemental material for Self-Management Exercise Regimen Versus Supervised Hand Therapy in Patients With Dorsal Dislocation or Hyperextension Injury With a Volar Plate Avulsion of the Proximal Interphalangeal Joint: The Randomized Controlled PINOT Study Supplemental material, sj-docx-2-han-10.1177_15589447261469595 for Self-Management Exercise Regimen Versus Supervised Hand Therapy in Patients With Dorsal Dislocation or Hyperextension Injury With a Volar Plate Avulsion of the Proximal Interphalangeal Joint: The Randomized Controlled PINOT Study by Dorien A. Salentijn, Priscilla Jawahier, Marit van Barreveld, Tosca Vrolijk-van Hengel, Niels Schep, Marcel G. W. Dijkgraaf, Mark van Heijl and Ruben N. van Veen in HAND

sj-docx-3-han-10.1177_15589447261469595 – Supplemental material for Self-Management Exercise Regimen Versus Supervised Hand Therapy in Patients With Dorsal Dislocation or Hyperextension Injury With a Volar Plate Avulsion of the Proximal Interphalangeal Joint: The Randomized Controlled PINOT Study Supplemental material, sj-docx-3-han-10.1177_15589447261469595 for Self-Management Exercise Regimen Versus Supervised Hand Therapy in Patients With Dorsal Dislocation or Hyperextension Injury With a Volar Plate Avulsion of the Proximal Interphalangeal Joint: The Randomized Controlled PINOT Study by Dorien A. Salentijn, Priscilla Jawahier, Marit van Barreveld, Tosca Vrolijk-van Hengel, Niels Schep, Marcel G. W. Dijkgraaf, Mark van Heijl and Ruben N. van Veen in HAND

sj-png-1-han-10.1177_15589447261469595 – Supplemental material for Self-Management Exercise Regimen Versus Supervised Hand Therapy in Patients With Dorsal Dislocation or Hyperextension Injury With a Volar Plate Avulsion of the Proximal Interphalangeal Joint: The Randomized Controlled PINOT Study Supplemental material, sj-png-1-han-10.1177_15589447261469595 for Self-Management Exercise Regimen Versus Supervised Hand Therapy in Patients With Dorsal Dislocation or Hyperextension Injury With a Volar Plate Avulsion of the Proximal Interphalangeal Joint: The Randomized Controlled PINOT Study by Dorien A. Salentijn, Priscilla Jawahier, Marit van Barreveld, Tosca Vrolijk-van Hengel, Niels Schep, Marcel G. W. Dijkgraaf, Mark van Heijl and Ruben N. van Veen in HAND

sj-png-2-han-10.1177_15589447261469595 – Supplemental material for Self-Management Exercise Regimen Versus Supervised Hand Therapy in Patients With Dorsal Dislocation or Hyperextension Injury With a Volar Plate Avulsion of the Proximal Interphalangeal Joint: The Randomized Controlled PINOT Study Supplemental material, sj-png-2-han-10.1177_15589447261469595 for Self-Management Exercise Regimen Versus Supervised Hand Therapy in Patients With Dorsal Dislocation or Hyperextension Injury With a Volar Plate Avulsion of the Proximal Interphalangeal Joint: The Randomized Controlled PINOT Study by Dorien A. Salentijn, Priscilla Jawahier, Marit van Barreveld, Tosca Vrolijk-van Hengel, Niels Schep, Marcel G. W. Dijkgraaf, Mark van Heijl and Ruben N. van Veen in HAND
