# Supplementary material for: Self-Management Exercise Regimen Versus Supervised Hand Therapy in Patients With Dorsal Dislocation or Hyperextension Injury With a Volar Plate Avulsion of the Proximal Interphalangeal Joint: The Randomized Controlled PINOT Study
Source: Hand (N Y). 2026 Aug 18:15589447261469595. Online ahead of print. doi: 10.1177/15589447261469595 (PMC13486395; doi:10.1177/15589447261469595)
Supplement: Supplementary file 1 [file sj-docx-1-han-10.1177_15589447261469595.docx]

**SUPPLEMENT**

Figure S1. Participant instructions at baseline

Figure S2. Mean MHQ-scores at baseline, 6 weeks and 3 months by PP treatment group Table S1. Table S1. Baseline characteristics ITT analysis set per treatment group

Table S2. Baseline characteristics PP analysis set per treatment group
