# Supplementary material for: Self-Management Exercise Regimen Versus Supervised Hand Therapy in Patients With Dorsal Dislocation or Hyperextension Injury With a Volar Plate Avulsion of the Proximal Interphalangeal Joint: The Randomized Controlled PINOT Study
Source: Hand (N Y). 2026 Aug 18:15589447261469595. Online ahead of print. doi: 10.1177/15589447261469595 (PMC13486395; doi:10.1177/15589447261469595)
Supplement: Supplementary file 2 [file sj-docx-2-han-10.1177_15589447261469595.docx]

**Table S1. Baseline characteristics ITT analysis set per treatment group***

| **Characteristics** | **Self-management**  **exercise regimen**  (n=105) | **Supervised**  **hand-therapy**  (n=106)** |
| --- | --- | --- |
| Age  18-34 years  35-64 years  ≥ 65 years | 55 (52)  43 (41)  7 (7) | 57 (54)  42 (40)  7 (7) |
| Gender (female) | 37 (35) | 46 (44) |
| Type of injury (complete dorsal dislocation) | 42 (40) | 42 (40) |
| Injured hand (right) | 52 (50) | 56 (53) |
| Dominant hand (right) | 90 (86) | 94 (90) |
| Dominant hand injured | 51 (49) | 51 (49) |
| Cause of injury |  |  |
| Sports | 52 (50) | 50 (48) |
| Work | 5 (5) | 4 (4) |
| Other | 48 (46) | 51 (49) |
| Study site  I  II  III  IV  Parkinson | 62 (59)  29 (28)  14 (13)  0 (0)  0 (0) | 64 (60)  25 (24)  16 (15)  1 (1)  0 (0) |
| Osteoporosis | 4 (4) | 1 (1) |
| Diabetes | 3 (3) | 2 (2) |
| Carpal tunnel syndrome (untreated) | 0 (0) | 2 (2) |
| History of operations of the affected finger | 0 (0) | 1 (1) |
| History of operations of the contralateral finger | 2 (2) | 3 (3) |
| Smoker | 21 (20) | 22 (21) |
| Alcoholism | 33 (31) | 21 (20) |
| Chronic use of corticosteroids | 0 (0) | 2 (2) |
| Chronic use Vitamin D | 23 (22) | 19 (18) |
| Chronic use Calcium suppletion | 4 (4) | 4 (4) |
| Chronic use Bisphosphonates | 0 (0) | 1 (1) |
| Hormone suppletion use | 5 (5) | 3 (3) |
| Health literacy (inadequate)^ | 8 (8) | 10 (10) |
| MHQ score, pooled mean (SD)^^ | 67.7 (15.4) | 67.0 (14.4) |

*count (%), unless otherwise stated. **For one immediate drop-out following randomisation to supervised hand-therapy only study site, type of injury, and age was registered. ^N_SMER_ =99, N_SHT_ =98. ^^Originally available: N_SMER_ =103, N_SHT_ =104.
