# Supplementary material for: Self-Management Exercise Regimen Versus Supervised Hand Therapy in Patients With Dorsal Dislocation or Hyperextension Injury With a Volar Plate Avulsion of the Proximal Interphalangeal Joint: The Randomized Controlled PINOT Study
Source: Hand (N Y). 2026 Aug 18:15589447261469595. Online ahead of print. doi: 10.1177/15589447261469595 (PMC13486395; doi:10.1177/15589447261469595)
Supplement: Supplementary file 3 [file sj-docx-3-han-10.1177_15589447261469595.docx]

**Table S2 . Baseline characteristics PP-population per treatment group***

| **Characteristics** | **Self-management**  **exercise regimen**  (n=85) | **Supervised**  **hand-therapy**  (n=79) |
| --- | --- | --- |
| Age  18-34 years  35-64 years  ≥ 65 years | 48 (57)  34 (40)  3 (4) | 41 (52)  32 (41)  6 (8) |
| Gender (female) | 31 (36) | 35 (44) |
| Type of injury (complete dorsal dislocation) | 34 (40) | 33 (42) |
| Injured hand (right) | 40 (47) | 50 (51) |
| Dominant hand (right) | 71 (84) | 72 (91) |
| Dominant hand injured | 38 (45) | 35 (44) |
| Cause of injury |  |  |
| Sports | 45 (53) | 40 (51) |
| Work | 4 (5) | 3 (4) |
| Other | 36 (42) | 36 (46) |
| Study site  I  II  III  IV  Parkinson | 57 (67)  19 (22)  9 (11)  0 (0)  0 (0) | 52 (66)  15 (19)  11 (14)  1 (1)  0 (0) |
| Osteoporosis | 4 (5) | 1 (1) |
| Diabetes | 1 (1) | 2 (3) |
| Carpal tunnel syndrome (untreated) | 0 (0) | 1 (1) |
| History of operations of the affected finger | 0 (0) | 1 (1) |
| History of operations of the contralateral finger | 1 (1) | 3 (4) |
| Smoker | 14 (17) | 17 (22) |
| Alcoholism | 29 (34) | 18 (23) |
| Chronic use of corticosteroids | 0 (0) | 1 (1) |
| Chronic use Vitamin D | 19 (22) | 14 (18) |
| Chronic use Calcium suppletion | 4 (5) | 2 (3) |
| Chronic use Bisphosphonates | 0 (0) | 1 (1) |
| Hormone suppletion use | 5 (6) | 2 (3) |
| Health literacy (inadequate)^ | 7 (9) | 7 (9) |
| MHQ score, mean (SD) | 68.5 (14.8) | 67.9 (13.1) |

*count (%), unless otherwise stated. ^N_SMER_=81, N_SHT_=75.
